# Curcumin as an effective suppressor of miRNA expression in patients with knee osteoarthritis

**DOI:** 10.22038/AJP.2021.19380

**Published:** 2022

**Authors:** Mahdi Atabaki, Zhaleh Shariati-Sarabi, Jalil Tavakkol-Afshari, Ali Taghipour, Mahmood Reza Jafari, Amin Reza Nikpoor, Mojgan Mohammadi

**Affiliations:** 1 *Clinical Immunology Research Center, Zahedan University of Medical Sciences, Zahedan, Iran*; 2 *Rheumatic Diseases Research Center, Mashhad University of Medical Sciences, Mashhad, Iran*; 3 *Immunology Research Center, Mashhad University of Medical Sciences, Mashhad, Iran*; 4 *Department of Epidemiology, Social Determinants of Health Research Center, School of Health, Mashhad University of Medical Sciences, Mashhad, Iran*; 5 *Nanotechnology Research Center, Pharmaceutical Technology Institute, Mashhad University of Medical Sciences, Mashhad, Iran*; 6 *Molecular Medicine Research Center, Hormozgan Health Institute, Hormozgan University of Medical Sciences, Bandar Abbas, Iran*

**Keywords:** Curcumin, MiR-146, MiR-15, MiR-138, MiR-16, Osteoarthritis

## Abstract

**Objective:**

Osteoarthritis is the most common disease in the group of joint diseases, and its incidence is directly related to aging. Given the anti-inflammatory effects of curcumin as an active ingredient of turmeric, we aimed to investigate the effects of this compound in a new curcumin nanomicelle formula named SinaCurcumin^®^ on the expression of microRNAs (miRNAs) involved in immune responses of patients with osteoarthritis.

**Materials and Methods:**

We divided 30 patients with osteoarthritis into two groups namely, nano curcumin-receiving (15 patients) and placebo-receiving (15 patients) and we studied them for 3 months. The Iranian Registry of Clinical Trials (IRCT) approved our study with the IRCT registry No. IRCT20151028024760N4. We evaluated the rates of the expression of microRNAs 146, 155, 16, and 138 employing SYBR Green Real-Time PCR method.

**Results:**

The expression of miRNAs 155, 138, and 16 revealed a significant reduction in the curcumin-receiving group (p=0.002, p=0.024 and p=0.0001 respectively).

**Conclusion:**

Our research data indicated that the consumption of curcumin in patients with osteoarthritis could affect the immune system partially via altering the expression of microRNAs and cytokines.

## Introduction

Osteoarthritis (OA) as the most frequent form of arthritis (Sakkas and Platsoucas, 2007 ▶) is a destructive and multifactorial joint disease affecting approximately 3.8% of the world's population. It is also the most common cause of inability to perform various activities in people (Li et al., 2017 ▶). The characteristics of this disease are variable, but pain, joint deformity, inflammation of the synovial membrane, dryness and progressive destruction of joints, and reduced range of motion are its prominent symptoms (Li et al., 2017 ▶; Zhu et al., 2020 ▶). Factors, such as age, gender, genetics of the individual, overweight, and immune system functions, play a crucial role in causing the disease (Zhu et al., 2020 ▶). Enzymes like matrix metalloproteinases (MMPs) are usually produced owing to responses of chondrocytes to physical damage, leading to stimulation of further damage to the joints. The release of joint components causes inflammatory reactions and induces innate and adaptive immune system responses. In such conditions, the infiltration of T and B-lymphocytes has been confirmed in the patients’ synovial tissue (Haseeb and Haqqi, 2013 ▶; Mehana et al., 2019 ▶). T Lymphocytes are the most important cells involved in the pathogenesis of the disease that play roles in the progression of the disease. T helper 17 (Th17) cells and CD4^+^ T cells are involved in this process (Lurati et al., 2015 ▶). Treg lymphocytes are CD4^+^CD25^+^CD127^-^FOXP3^+^ that regulate immune responses and inhibit inflammatory processes by secreting Interleukin 10 (IL-10) and Transforming Growth Factor β (TGF-β) cytokines (Gol-Ara et al., 2012 ▶; Luckheeram et al., 2012 ▶). Concerning the functions of regulatory T cells, a study indicated that the number of these cells was increased in the patients' synovial fluid, while the level of IL-10 production as an inhibitory cytokine was decreased in patients with osteoarthritis, demonstrating the impaired inhibitory mechanisms of immune responses in the disease process (Li et al., 2016 ▶).

Curcumin as a polyphenolic turmeric (*Curcuma longa*)-derived compound has been widely considered in recent research for its anti-inflammatory and anti-proliferative properties. Furthermore, it has been administered for the treatment of diseases such as cardiovascular diseases, diabetes and autoimmune diseases (Zhang and Zeng, 2019 ▶). Several studies have revealed curcumin immunomodulatory effects on pathways regulating immune responses (Atabaki et al., 2020 ▶). The safety and nontoxic properties of this agent have been reported by the Food and Drug Administration of the United States, even after daily administration of up to 8000 mg for three months (Atabaki et al., 2020 ▶).

MicroRNAs are a group of post-transcriptional regulatory factors causing degradation of mRNA or inhibiting its translation into protein (Su Huang et al., 2017 ▶). These molecules can be organized in different components inside the cell, such as endoplasmic reticulum, mitochondria and nucleus, and they are secreted from the cell into different body fluids like plasma through the exosome (Sondag and Haqqi, 2016 ▶). RNA polymerase II enzyme transcribes the microRNA gene. The first transcribed structure has a hairpin structure on its 3´ side that is removed by the Drosha enzyme. This version is transferred from the nucleus to the cell cytoplasm by Exportin 8 and is detected by the Dicer enzyme, which is a RNase III, and is finally converted to a mature microRNA (Sondag and Haqqi, 2016 ▶). These factors are a group of small non-coding RNAs with a size of approximately 17 to 22 nucleotides that bind the 3´ end of mRNA, and effectively control the process of transcription and expression of approximately 60% of human genes. Many aspects of immune system responses and homeostasis are regulated through the function of these molecules (Merkenschlager, 2014 ▶; Phuah and Nagoor, 2014 ▶; Ma et al., 2015 ▶; Afshar et al., 2019 ▶). This study aimed to evaluate the properties of curcumin in a new nanomicelle formula on the expression of microRNAs involved in the immune system responses of patients with osteoarthritis.

## Materials and Methods

**Study design and ethics**

**Figure 1:**
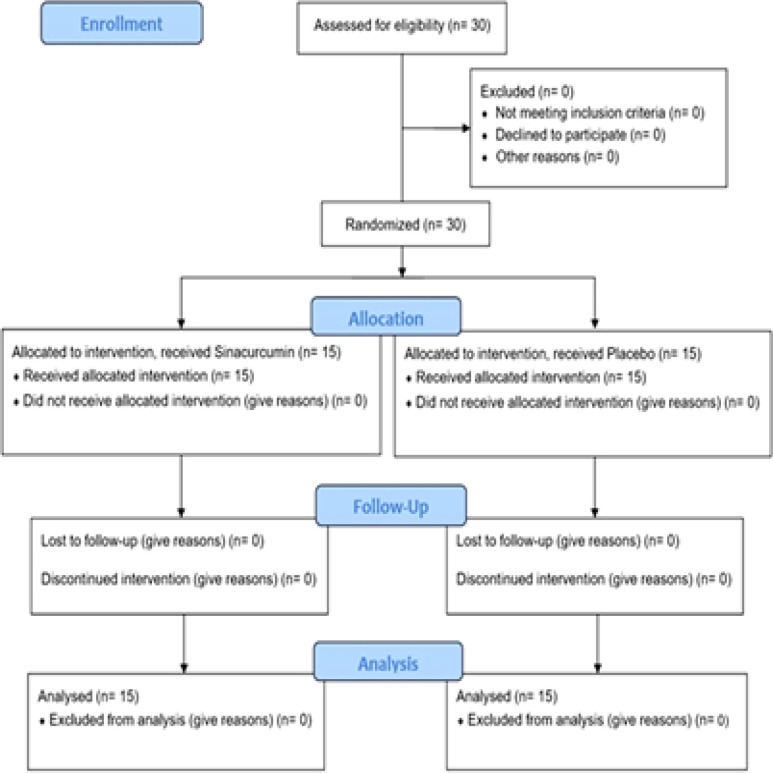
Consort flow diagram

We conducted our study as a double-blind randomized clinical trial in Mashhad, capital of Khorasan Razavi Province, Iran. The Iranian Registry of Clinical Trials (IRCT) approved our trial by the IRCT registry No. IRCT20151028024760N4. In addition, we registered our study in Clinicaltrials.gov (NCT03715140). The Ethics Committee of Mashhad University of Medical Sciences also approved the present study (IR.MUMS.MEDICAL.REC.1397.118). As a statement of ethical principles in medical investigation, all participants completed written consent forms according to the Declaration of Helsinki. This trial followed the CONSORT statement and Figure 1 explains it. For further details, please see our recently published article (Atabaki et al., 2020 ▶). 

**The inclusion criteria**

Patients suffering from osteoarthritis with a pain period of more than 6 months in their knee, age of 40 to 55 years, grade II or III of osteoarthritis based on the Kellgren-Lawrence (K-L) index, no history of joint surgery, no long-term use of anti-inflammatory drugs, and no history of underlying diseases such as diabetes, osteonecrosis, gout, or kidney disorders, and with a body mass index (BMI) of less than 30, were enrolled to the trial. 

**Patient allocation and randomization**

The permuted block randomization method was used to randomly allocate the patients to each group of study, and an independent person arranged the randomization sequence through a computerized random sequence creator. A randomization sequence was allocated to each qualified participant in 1:1 ratio to consume either curcumin or placebo, and a drug with a resembling sequence was ordered. An independent individual, who managed SinaCurcumin® or placebo as per the assigned randomization sequence, performed the blinding of the patients and the physician.

**Preparation and administration of curcumin and placebo**

The nanomicelle form of curcumin named SinaCurcumin^®^, which contains 80 mg of the curcumin (IRC: 1228225765), is produced by Exir Nano Sina Corporation (Tehran, Iran). We purchased the drug and the placebo from this corporation. SinaCurcumin^®^ 80 mg and placebo were administered to the intervention and the placebo group, respectively, both taking one capsule daily for a period of three months. Moreover, 50 mg sodium diclofenac in an anti-pain dosage was administered to all patients as a conventional therapy.

**Demographic and anthropometric data and visual analogue scale score of patients with knee OA**

In agreement with the standard method, the patients’ height and weight were evaluated for all participants at the end of an overnight fasting. For each person, the Body Mass Index (BMI) was assessed by the weight (kg) divided by the height (m) squared. Based on age and gender, the patients were matched with each other in two different sets. To measure and evaluate the patients’ pain intensity and severity, the VAS index was used (0 for no pain to 10 for the maximum pain). Our latest published article presents the related data (Atabaki, Shariati-Sarabi et al. 2020 ▶).

**Blood specimen preparation**

Before and after three months of conducting the study, 2 ml blood with EDTA was obtained from all 30 participants to assess the microRNA expression level.

**Extraction of microRNA, synthesis of cDNA, and conducting SYBR Green Real-Time PCR (qRT-PCR)**

We utilized the SanPrep Column MicroRNA Miniprep Kit (Biobasic, Canada) according to the company's directions to extract microRNA from the whole blood samples. We measured the concentration and purity of microRNA and cDNA employing a NanoDrop Spectrophotometer (Thermo Fisher Scientific, USA). We also used the Bon-Mir RT Kit (Bonyakhteh, Tehran, Iran) to synthesize cDNA according to the manufacturer's instructions. Briefly, 5 μl of microRNA, 1 μl of rATP, 2μl of poly A polymerase buffer and 0.1 μl of poly A polymerase enzyme were added to a tube, and using DNAs, RNAs free water, total volume was increased to 20 μl. Poly A polymerization reaction was performed by 37^ο^C incubation for 30 min and 65^ο^C incubation for 20 min. Then, 10 μl of ploy adenylated microRNA, 1 μl of BON-RT adaptor primer and 2 μl of DNAs, RNAs free water were added to another tube and incubated at 75^ο^C for 5 min. Finally, cDNA synthesis was performed by addition of 1 μl of RT enzyme, 2 μl of dNTP, and 4μl of RT buffer to the tube and by incubation at 25^ο^C for 10 min, at 42^ο^C for 60 min, and at 70^ο^C for 10 min.

**Table 1:** Sequences for forward primers of targeted microRNAs and *SNORD47* (the housekeeping gene)

**Primer Length (bp)**	**Forward primer sequence (5´ 3´)**	**NCBI-/miRBase-mature sequence accession**	**MicroRNA**
20	GCTGAGAACTGAATTCCATG	MIMAT0000449	miR146a-5p
18	GCTAATCGTGATAGGGGT	MIMAT0000646	miR155-5p
19	GCTGGTGTTGTGAATCAGG	MIMAT0000430	miR138-5p
19	GGCATAGCAGCACGTAAAT	MIMAT0000069	miR16-5p
18	ATCACTGTAAAACCGTTC	X96647	SNORD47

We purchased the specific forward primers for each targeted microRNA and the universal reverse primer from the Stem Cell Technology Research Center (Bonyakhteh, Tehran, Iran). Table 1 displays the sequences of the forward primers of targeted microRNAs and Small Nucleolar RNA, C/D Box 47 (*SNORD47*) used as the housekeeping gene.

We performed the SYBR Green Real-Time PCR (RT-PCR) reaction employing the Rotor Gene 6000 device (Qiagen, USA) to evaluate the expression of microRNAs. *SNORD47* was recruited as an internal control gene. We performed all reactions in duplicate and used the 2^-ΔΔCT^ method to analyze the gene expression. In a 10 μl total volume reaction, 5 μl SYBR Green Mastermix, 0.5 μl of specific forward primer, and universal reverse primer (10 pmol/µl), and 4 μl cDNA were considered. SYBR Green Real-time PCR cycling conditions for all microRNAs were 95°C for 10 min followed by 40 cycles of 10 sec at 95°C, 30 sec at 60°C, and 20 sec at 72°C.

**Statistical analysis**

After collecting the relevant data, we entered the data into the SPSS statistics version 21 software. We used the Kolmogorov-Smirnov test to check the data normality. We applied the t-test for normal data, and used the unpaired t-test to check if the data were non-normal. Finally, a p-value less than 0.05 was considered significant.

## Results

**Expression level of microRNA genes**

**Figure 2:**
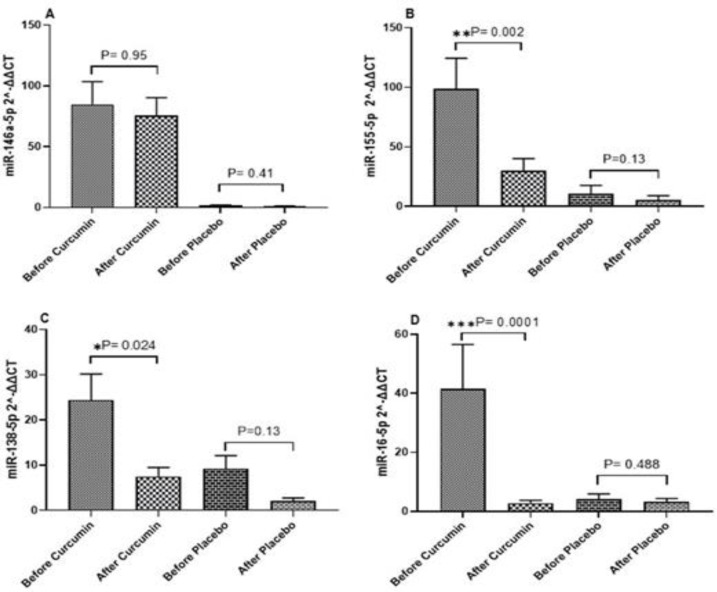
Expression level of targeted microRNA (2^-ΔΔCT) before and after the intervention

Three months after conducting the study, the Mir-146 gene expression level in the group of curcumin-receiving patients indicated an insignificant decrease (p=0.96). The expression level of this gene in placebo-receiving patients also exhibited an insignificant reduction (p=0.41). The Mir-138 gene expression level showed a significant decrease in the curcumin-receiving patients (p=0.024), but an insignificant decrease in placebo-receiving patients (p=0.13). The Mir-155 gene expression level indicated a significant decrease in the curcumin-receiving patients (p=0.002), but an insignificant decrease in placebo-receiving patients (p=0.13). The Mir-16 gene expression level showed a significant decrease in the curcumin-receiving patients (p=0.0001), and an insignificant decrease in placebo-receiving patients (p=0.48). Figure 2 present the data related to the expression level alteration of four targeted genes in this trial. 

## Discussion

**Clinical and para clinical criteria**

In our present study, in the Sinacurcumin® group and placebo group, a significant decline and a non-significant rise were detected in the VAS score, respectively. Data presented in a review article showed that in OA patients, curcumin affected the pain and quality of life considerably (Onakpoya et al., 2017). It was indicated that curcumin had similar performance to sodium diclofenac on the VAS score in OA patients (Shep et al., 2019 ▶). It has been demonstrated that a combination of curcumin and sodium diclofenac significantly improves the intensity of pain in OA patients (Shep et al., 2019 ▶). The results of a trial revealed that the knee VAS score in OA participants was decreased in the curcumin group after an eight-week period of curcumin prescription (Nakagawa et al., 2014 ▶). Our study results are compatible with those described in the cited studies. In addition, the data of a study on rheumatoid arthritis patients under curcumin treatment revealed that the VAS score was significantly decreased after three months of their treatment (Amalraj et al., 2017 ▶); the results of this study were similar to ours. 

The ESR and CRP level are associated with the OA progress and its clinical characteristics (Hanada et al., 2016 ▶). The CRP level was significantly decreased in our trial in the intervention group, but the ESR level in the intervention group exhibited insignificant changes at the 

end of our current study. A 6-week follow-up period of one study, demonstrated a significant reduction in the CRP level and an insignificant alteration in the ESR level after curcumin administration (Rahimnia et al., 2015 ▶). Panahi et al. reported that curcumin reduced the CRP concentration in OA patients (Panahi et al., 2015 ▶). In line with our current trial, Belcaro et al. described that the CRP level of OA patients was significantly declined owing to prescription of curcumin (Belcaro et al., 2010 ▶). 

**SYBR Green** **Real-Time PCR (qRT-PCR)**

Jumonji and AT-rich interaction domain containing 2 (*Jurid2*) gene is a member of the Jumonji family that works due to altering the chromatin by removing the methyl group from the lysine bases and by inducing the gene expression. This gene is an important target for microRNA-155. Jurid2 is related to the polycomb-repressive complex 2 (PRC2) factor that is expressed in embryonic stem cells (ESC) and plays its role in the differentiation of these cells into T-helper (Th) cells. Furthermore, Jurid2 plays its controlling role in the production and secretion of cytokines, such as IL-9, IL-22, and IL-10, which are essential elements in the differentiation of Th17 cells. MicroRNA-155 plays its role by controlling Jurid2 performance in differentiation of Th17 cells and secretion of cytokines from these cells (Merkenschlager, 2014 ▶). 

Activation of microRNA-155 can induce B lymphocytes and antigen presenting cells (APCs) functions and control the functions of Th and Treg cells (Merkenschlager, 2014 ▶). Inhibition of this regulatory agent can control production of inflammatory cytokines and activities of various cells involved in the inflammatory process, and lead to inhibition of inflammation (Zhang et al., 2017 ▶).

MicroRNA-146 is an important microRNA expressed in the cartilage of patients with osteoarthritis, and is related to the pain score in these patients. At the early stages of joint destruction, IL-1 increases the expression of this microRNA that plays a key role in inhibiting catabolic elements due to the interleukin-1 receptor-associated kinase 1 (IRAK1) as well as the tumor necrosis factor receptor associated factor 6 (TRAF6). On the contrary, the expression of this microRNA decreases at the late stages of the joint destruction phase and it is associated with the progressive joint destruction (Kopanska et al., 2017 ▶). MicroRNA-146 inhibits inflammatory processes and reduces inflammation by blocking the messaging pathways associated with TRAF6, IRAK1, interferon regulatory factor (IRF) 3, and IRF5 (Testa et al., 2017 ▶; Chen et al., 2018 ▶). 

One important pathway associated with inflammation is the NFkB-mediated signaling pathway, which is inhibited by microRNA-146 (Hsu et al., 2017 ▶). It has been found that the expression of inflammatory cytokines such as IL-17, and RAR-related orphan receptor gamma t (RORγt) transcription factor, increases in the absence of this microRNA (Li et al., 2017 ▶). Moreover, studies have found that stimulation of microRNA-146 expression decreases the response of chondrocytes to TGF-β cytokine, and increases apoptosis in these cells (Nugent, 2016 ▶). It can be concluded that the important factor that effectively determines the activities and function of a microRNA is the microenvironment around it that partially justifies its different behavior.

MicroRNA-16 is an effective molecule in the induction of inflammatory reactions. It has a high expression level in inflammatory conditions and its expression is directly associated with the expression of the RORγt transcription factor. MicroRNA-16 stimulates the RORγt transcription factor and increases the Th-17 cell differentiation by increasing the expression of TNF-α. Furthermore, it decreases the expression of the forkhead box P3 (FOXP3) transcription factor and the Treg cell differentiation (Wu et al., 2016 ▶). However, the findings have indicated that the expression of this microRNA increases in the cartilage of patients with osteoarthritis. TGF-β cytokine plays a role in the proliferation of chondrocyte cells and sends its message to the cell by SMAD3; therefore, it causes the proliferation of cartilage cells. The expression of SMAD3 decreases in patients with osteoarthritis owing to the inhibitory action of microRNA-16, thereby inhibiting the cell proliferation, increasing the expression of ADAMTS and metalloproteinase enzymes, and decreasing the expression of various components of the cellular matrix, such as collagen type II and Agricane. Thus, the disease conditions lead to a further destruction of cartilage (Li et al., 2015 ▶; Panagopoulos and Lambrou, 2018 ▶).

MicroRNA-138 is an important regulatory factor connected with the process of apoptosis, cell differentiation, and induction of stem cell differentiation. This factor reduces the expression of important components associated with the extracellular matrix of cartilage by inhibiting two molecules, Sp-1 transcription factor, and Hypoxia-inducible factor (HIF) 2α. Owing to this function, inhibition of microRNA-138 can lead to the gene expression of the extracellular matrix components of cartilage, maintain the cartilage homeostasis, and have a therapeutic function in patients with OA (Seidl et al., 2016 ▶). It has been found that the expression of microRNA-138 increases in patients with osteoarthritis; therefore, the interaction between this microRNA with the IL-1β gene destroys the extracellular matrix of cartilage in OA patients. Moreover, microRNA-138 regulates the gene expression of FOXC1, and increases the matrix degradation, along with the function of IL-1β (Kopanska et al., 2017 ▶).

It has been discovered that microRNA-155 plays a crucial role in controlling the antibody isotype switching by regulating the activation-induced cytidine deaminase (AID) enzyme function. This microRNA is necessary in the response of B lymphocytes to thymus-dependent and thymus-independent antigens; and the B lymphocyte count decreases in germinal centers in cases with lower expression of microRNA-155. On the contrary, when the expression of microRNA-155 increases, the B lymphocyte count increases in germinal centers, thereby increasing the level of antibody production. In addition, B lymphocytes with defective microRNA-155 functions are unable to perform antibody isotype switching and produce high-affinity IgG1 antibody (Zheng et al., 2018 ▶). 

MicroRNA-146 plays an essential function in the process of B lymphocytes differentiation in the marginal zone of the spleen, and it has been found that the defect in the performance or lack of this microRNA reduces the production of B cells (King et al., 2016 ▶). 

According to the results of the present study, curcumin decreases the expression of microRNAs-155 and 146, and it can be concluded that this component regulates the B lymphocyte functions in patients with OA, thereby reducing the patients’ clinical symptoms.

In our present trial, the expression level of microRNA-138, 155, and 16 was significantly decreased in curcumin-receiving patients after a three-month period. The expression of microRNA-146 exhibited a non-statistically significant decrease in this group. In the placebo group, the expression level of all four microRNAs was decreased insignificantly.

Ma et al. assessed the function of curcumin in rats stimulated with lipopolysaccharide (LPS) by intraperitoneal injections. Their results indicated that curcumin decreased the expression of microRNA-155, and controlled the inflammatory conditions by inhibiting the signaling pathway of PI3K-AKT in macrophages (Ma et al., 2017 ▶).

It has been reported that microRNA-16 decreases the expression of anti-apoptotic BCL-2 protein and stimulates apoptosis in eukaryotic cells. Curcumin decreases the expression of BCL-2 by increasing the expression of microRNA-16, thereby inhibiting cellular apoptosis (Momtazi et al., 2016 ▶).

The effects of curcumin on the expression of microRNA have been evaluated in different diseases. Studies investigating the effect of curcumin on thymus carcinoma cells indicate that the survival of these cells, their migration, and their invasion decreased due to the inhibitory effect of curcumin on the expression of microRNA-27a (Han et al., 2020 ▶). Curcumin can decline the expression of the matrix metalloproteinase (MMP) 9 enzyme by activating and increasing the expression of microRNA-365 and as a result, bone destruction was reduced in rats (Li et al., 2015 ▶). 

Given that most studies examining curcumin properties on different microRNAs had focused on cell lines and cancer, we had no sufficient references to compare the results of our study on the function of curcumin on the expression of microRNAs to them. Based on the results of various studies investigating the functions of microRNAs, the study of these regulatory molecules in patients can be highly helpful as an index to measure disease activities and to investigate disease progression or control. These factors are good guidelines to adopt a treatment strategy for OA patients’ symptoms.

The results of this trial present confirmatory records of the significant immunomodulatory properties of the nanomicelle form of curcumin on T lymphocytes and B lymphocytes in OA patients owing to its effects on the function of microRNAs. Daily administration of 80 mg curcumin can considerably decrease pain and inflammation in OA patients and ameliorate the activity and severity of the disease possibly due to its regulatory properties on microRNAs. Recent studies have demonstrated that curcumin has no side effects (Atabaki et al., 2020 ▶); thus, it is recommended as a possible alternate herbal treatment with appropriate anti-pain and anti-inflammatory properties in patients suffering from OA.

## Conflicts of interest

The authors have declared that there is no conflict of interest.
